# Work Experience of Chinese Male Nurses Based on the Job Demands-Resources Model: A Cross-Sectional Study

**DOI:** 10.1155/2023/6486195

**Published:** 2023-02-21

**Authors:** Yiming Gao, Jian Li, Zihui Xie, Xiangyu Zhao, Di Zhao, Qing Wang, Miao Zhou, Ping Li

**Affiliations:** ^1^Department of Health Psychology, School of Nursing and Rehabilitation, Cheeloo College of Medicine, Shandong University, Jinan, Shandong 250012, China; ^2^Shandong Provincial Hospital Affiliated to Shandong First Medical University, Jinan, Shandong 250021, China

## Abstract

**Aim:**

This study aimed to quantitatively compare the effects of perceived social support, resilience, and task load on occupational burnout and flow at work in male nurses, as well as the complex relationships among these variables.

**Background:**

Male nurses play a vital role in the healthcare system. However, little is known about the key factors that may improve the work experience of male nurses.

**Methods:**

A cross-sectional study was conducted from June to December 2021. A convenience sample of 356 male nurses completed measures of general information, burnout, flow at work, perceived social support, resilience, and task load. Dominance analysis and network analysis were used to explore the associations between the variables studied.

**Results:**

Among the variables studied, perceived social support most strongly predicted burnout, while perceived social support and resilience had equal predictive weights in flow at work. The network analysis found that resilience was the bridge indicator with the strongest connections to other variables.

**Conclusion:**

The interventions focused on the perceived social support and resilience of male nurses could help improve their work experience. *Implications for Nursing Management*. Nursing administrators should give male nurses more support and understanding to reduce burnout at work. At the same time, administrators can conduct psychological lectures to improve male nurses' resilience, thereby increasing their flow at work. Providing challenging tasks that match male nurses' skills could also help improve their work experience.

## 1. Introduction

The latest data from the National Bureau of Statistics of China in 2022 showed that the number of registered nurses (RNs) in China had reached 5.02 million, of which 150,000 were male nurses. In the case of the shortage of nursing human resources, male nurses had unique advantages. For example, they were physically stronger, good at dealing with complex devices, and handled problems more rationally [1]. However, since the time of Nightingale, nursing is often considered a domain of women [2, 3]. Male nurses have a lower sense of professional identity and higher turnover intention due to many factors such as traditional beliefs, self-concept, social status, and income [4, 5]. How to attract more male nurses to stay in the nursing profession has become a concern for nursing administrators.

Previous studies showed that nurses' work experience, such as burnout, significantly predicted their turnover rate. For example, a longitudinal study of 1688 nurses showed an 11.62% increase in the likelihood of turnover for a unit increase in the emotional exhaustion dimension of burnout [6]. Occupational burnout, as one of the most concerned work experiences in the field of occupational health, refers to a state of emotional exhaustion, cynicism, and reduced personal accomplishment experienced by individuals in the human-serving professional field [7]. Actually, burnout continues to be a persistent and concerning problem for male nurses. A previous study showed that the burnout rate of male nurses in China was as high as 59.1%–69.1% [8]. Occupational burnout not only affects the physical and mental health of individuals [9] but also causes a series of adverse effects, such as the decline in nursing quality and doctor-patient conflict [10]. These further increase the work instability of male nurses. Although many studies have been conducted globally to reduce nurses' burnout, the focus on male nurses is still lacking.

Flow at work (FaW), as a positive work experience, has received increasing attention in recent years with the flourishing development of positive psychology. Bakker proposed that FaW was a short-term peak experience at work characterized by absorption, work enjoyment, and intrinsic work motivation [11]. According to the broaden-and-build theory, this positive state leads to a series of positive outcomes for the individual at work, including positive experiences, thoughts, and feelings that promote optimal functioning and well-being [12]. Some studies were conducted to explore the positive effects of flow. For example, FaW was found to be associated with higher performance [13], more positive emotions, and lower turnover intentions [14], thus playing an important role in improving the professional identity and attitudes of nurses [15, 16]. However, most of the recent studies used the entire nursing team as the research object, and the attention on male nurses' FaW was lacking. Hence, this study aimed to explore the key factors that enhanced the work experience of male nurses from the perspectives of burnout and FaW.

The job demands-resources (JD-R) model is a theoretical framework most widely used in occupational health research. The main assumption of the model was that each occupation has factors associated with job stress, which can be classified into two categories: job demands and job resources. Previous studies found that the JD-R model could predict the experiences of burnout and work engagement [17]. As a job demand in the clinical nursing field, most previous studies found that the task load could positively predict burnout [18, 19]. Regarding job resources, previous research showed that the perceived social support (PSS) and resilience could improve the work experience of nurses. As an external resource, PSS refers to an individual's subjective perceptions of support from other people [20], including family support, friends' support, and other supports (such as colleague support). PSS enables nurses to seek assistance to cope with stressors in the workplace [21], and can reduce burnout and enhance the flow experience [22, 23]. Resilience, as one of the internal coping resources, refers to the ability of an individual to recover from failure, adversity, uncertainty, or overpowering change [24]. Individuals with higher levels of resilience can overcome the negative effects of workplace adversity and challenges, resulting in less burnout and more flow experiences [25, 26].

However, little research has quantitatively compared the effect of the aforementioned variables on nurse burnout and FaW. The traditional multiple regression determines the relative importance of predictors, usually using standardized regression coefficients or *R*^2^ values. However, if the analyzed predictors are correlated, for instance, nurses with greater PSS demonstrate higher levels of resilience [21], and the regression analysis results tend to be dependent on the existing model. In addition, using standardized regression coefficients to determine the relative importance may exaggerate or reduce the effect of predictors [27]. The dominance analysis calculates the average direct effect of each variable (only considering the variable itself), the overall effect (depending on all predictive variables in the full model), and the partial effect (depending on all other predictive variables in the subset model). This method explains the relative weights of predictive variables while comparing all possible subset models. Therefore, it is more accurate and intuitive to determine the sequence of relative importance among predictors by using this method. In this context, the present study compared the relative predictive weights of PSS, resilience, and task load on the work experience of nurses using dominance analysis.

In this study, the dominance analysis was conducted based on the sum scores of self-report measures of the participants. However, both burnout and FaW, which represented the work experience of male nurses, and PSS and task load, which were predictor variables, all have different components (i.e., different dimensions). Therefore, exploring the relationships among variables only based on the total scores may still be problematic, as it may overlook different associations between variable dimensions and restrict the development of intervention methods. In recent years, network analysis has gained increased attention for its ability to provide inter-relationships among the factors [28]. In general, the network structure consists of nodes (variables of interest) and edges (connections between variables). It allows for studying the complexity of a selected structure. At the same time, it also provides centrality indices that characterize the nodes in the network, thus reflecting which nodes are more important in the network. Thus, on the one hand, using network analysis may contribute to the existing knowledge by elucidating the relationships between predictor variables (PSS, resilience, and task load) and work experience (burnout and FaW) at a component level. On the other hand, the bridge centrality strength index (i.e., the sum of the value of all edges connecting a specific node to other community nodes) for all nodes was estimated to find the bridge node, thus providing more references for the development of targeted interventions.

**Figure 1:**
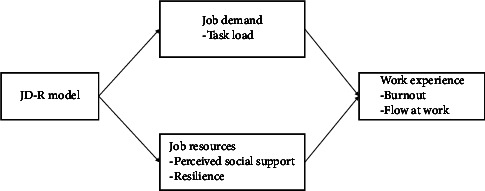
The conceptual map of study variables.

Taken together, the aim of this study was (1) to investigate levels of occupational burnout and FaW in male nurses, (2) to compare the relative predictive weights of job demands-resources-related variables (PSS, resilience, and task load) on the work experience of male nurses (occupational burnout and FaW) through dominance analysis, and (3) to further explore the complex relationship among multiple variables and identify the key variables in connecting the network using the network analysis while considering both burnout and FaW. The conceptual map of study variables is shown in Figure 1.

## 2. Materials and Methods

### 2.1. Design

This study adopted a cross-sectional design.

### 2.2. Sample

Participants were recruited from 10 public hospitals in Jinan, Shandong Province, China. The inclusion criteria were as follows: (a) having a Chinese RN license and (b) male nurses engaged in nursing work for ≥1 year. The study nurses or nurses on leave were excluded. In this study, a total of 402 electronic questionnaires were collected. After excluding the questionnaires with less than 5 min of response time, 356 questionnaires were finally included in the analysis. The sample size ranges up to 350, so high specificity, moderate sensitivity, and edge weights correlations can be properly seen in the network model [29].

### 2.3. Measures

The sociodemographic and work-related data were assessed with a researcher-designed questionnaire, mainly including age, marital status, child status, economic conditions, education level, work department, professional title, position, hospital level, work seniority, and the number of night shifts per month.

The Maslach Burnout Inventory-General Survey (MBI-GS) is a widely used burnout assessment tool [30]. This scale consisted of 15 items including the following 3 subdimensions: emotional exhaustion (five items), cynicism (four items), and personal accomplishment (six items). Each item was rated on a 7-point scale (0 = never and 6 = very frequent). The first two subdimensions were scored positively, while the personal accomplishment subdimension used reverse scoring. Higher scores indicated more severe occupational burnout. In this study, Cronbach's *α* was 0.854 for the entire scale and 0.964, 0.948, and 0.945 for emotional exhaustion, cynicism, and personal accomplishment, respectively.

This study used the work-related flow inventory (WOLF) revised by Zhu [31] to measure the FaW of nurses, which was a revision of the original version developed by Bakker [32]. The scale contained three subdimensions of FaW: absorption, work enjoyment, and intrinsic work motivation. The participants were asked to rate their agreement with each item on a 5-point Likert scale from 1 (strongly disagree) to 5 (strongly agree). Higher scores indicated higher FaW experiences. The Cronbach's *α* coefficients for the whole scale and subdimensions were 0.952, 0.908, 0.958, and 0.903, respectively, in the present study.

The Perceived Social Support Scale was used to measure the subjectively perceived levels of social support among male nurses [33]. This scale included three subdimensions of family support, friends' support, and other support, using a Likert 7-point scoring (1 = strongly disagree and 7 = strongly disagree). The total score ranged from 12 to 84, with higher scores indicating more PSS. The Cronbach's *α* coefficients for the whole scale and subdimensions in this study were 0.969, 0.936, 0.950, and 0.926, respectively.

The 10-item Connor F02D Davidson Resilience Scale (CD-RISC-10) was used to measure the resilience level of individuals [34]. This was a 5-point Likert scale, and each item was scored from 0 to 4 (0 = never, 1 = rarely, 2 = sometimes, 3 = often, and 4 = always). The total score was the sum of the scores of all items, and a higher total score indicated a higher level of resilience. In this study, the Cronbach's *α* for this scale was 0.965.

The task load was measured using the NASA-Task Load Index (NASA-TLX) scale proposed by Hart [35]. The original scale had six items. In this study, the revised Chinese version was used [36], which retained four items. Each item was a subdimension, namely, mental effort (ME), physical effort (PE), time pressure (TP), and effort level. Each item was scored from 0 to 20, represented by a line divided into 20 equal parts, with “low” and “high” marked at each end of the line. The higher the score, the heavier the task load. The Cronbach's *α* of the total scale in this study was 0.887.

### 2.4. Data Collection

Data collection for this study was conducted from June to December 2021. After explaining the purpose and methods of the study, approval was obtained from the nursing principal of each hospital surveyed. The survey host of “So Jump” was used for collecting the questionnaires online. Participants accessed the survey by scanning the QR code and clicking on the secure link.

### 2.5. Data Analysis

All the analyses were conducted with SPSS version 26.0 (IBM Corp, NY, USA) and *R* software version 4.2.0 (*R* Foundation for Statistical Computing, Vienna, Austria). Continuous variables were presented as means (*M*) and standard deviations (SD), while the categorical variables were described using frequencies (*N*) and percentages (%). Pearson's correlation analysis was used to analyze the correlations of study variables.

The dominance analysis [37] was developed by Budescu to compare the relative importance of predictors in multiple regression by examining the *R*^2^ values for all possible subset models (2^*P*^ − 1, where *P* is the number of predictors in the full model). This approach was used in the present study to compare the relative importance of PSS, resilience, and task load for occupational burnout and FaW. According to the theoretical basis of a dominance analysis, we conducted seven regression analyses to test all possible combinations of the predictor variables and obtained averaged ∆*R*^2^ values to compare the weight of the predictors.

In this study, we estimated a Gaussian graphical model (based on polychoric correlations) containing PSS (three nodes), resilience, task load (four nodes), occupational burnout (three nodes), and FaW (three nodes) to explore the connections between these nodes. A Gaussian graphical model is an undirected weighted network suitable for association analysis among continuous variables, in which edge weights can be understood as partial correlation coefficients (i.e., controlling the effects of all other items in the network). An extended Bayesian information criterion (EBIC) least absolute shrinkage and selection operator (LASSO) procedure was used for network modeling to obtain a more concise network. The network was visualized using the qgraph package. In the network structure, “edges” are the lines between nodes representing regularized partial correlations. Two nodes are connected by an edge when their partial correlation coefficient is not equal to zero. The thicker the edge, the stronger the association. For a deeper understanding of which indicator was most closely connected to other constructs, we subsequently estimated the bridge centrality strength for all nodes. In addition, we used the bootnet package of *R* software to calculate the correlation stability (CS) coefficient to evaluate the stability of the network. A previous study suggested that the CS-coefficient ≥0.5 indicated that the network model had better stability [38]. All variables on different scales were normalized to avoid the potential impact. The related codes are available in the Supplementary File.

All statistics were two-tailed, and *P* values <0.05 indicated statistically significant differences.

### 2.6. Ethical Considerations

The research ethics committee of the affiliated institution approved the study, and informed consent was obtained from all participants before questionnaire collection, ensuring that the responses were voluntary and anonymous.

## 3. Results and Discussion

### 3.1. Sociodemographic and Work Characteristics

A sample of 356 male nurses from 10 public hospitals who met the inclusion criteria was obtained. The participants were aged 20–53 years (*M* = 30.6; SD = 5.8). Among the participants, 7% were from surgical wards, 12.6% from medical wards, and 44.7% from an emergency or intensive care ward. The mean number of working years was 8.5 (range 1–32) for 356 male nurses, and they had an average of 7.2 (range 0–26) night shifts per month. More details are presented in Table 1.

### 3.2. Descriptive Statistics and Correlation Analysis

The scores of emotional exhaustion, cynicism, and low personal accomplishment for male nurses in this study were (9.76 ± 7.90), (6.09 ± 6.05), and (18.73 ± 9.63), respectively. The FaW score was (44.50 ± 10.98). As shown in Table 2, resilience, PSS, and task load were all negatively correlated with burnout (*r*_*s*_ = −0.164 to −0.432 and *P* < 0.01) and positively correlated with FaW (*r*_*s*_ = 0.155–0.516 and *P* < 0.01). Notably, all job demands-resources-related variables were correlated with one another (*r*_*s*_ = 0.259–0.741 and *P* < 0.01), indicating that the dominance analysis rather than multiple regression was needed to compare the predictive weight [39].

### 3.3. Dominance Analysis

Table 3 presents the results of the dominance analysis. The numbers in the table represent the average ∆*R*^2^ of each predictor when added to regression equations with different subsets of the other predictors. When the outcome variable was occupational burnout, PSS accounted for 14.6% of the average variance, making it the most important predictor. When the outcome variable was FaW, both PSS and resilience accounted for 18.1% of the average variance. In both analyses, the task load was the least significant predictor.

### 3.4. Network Analysis

**Figure 2:**
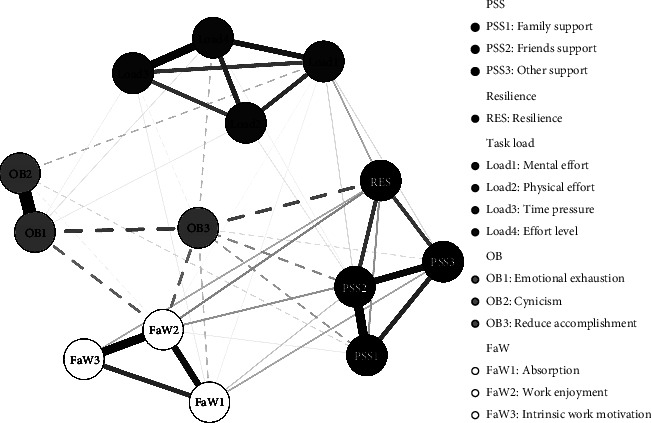
Visualization of the network (*n* = 356).

**Figure 3:**
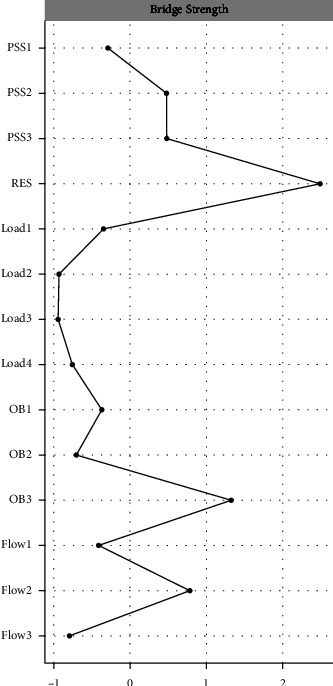
Bridge centrality strength of study variables (*n* = 356).

In the present study, the purpose of network analysis was to explore the association between different dimensions of variables and to further discover bridge nodes. Given the complexity of the network analysis approach and the purpose of this study, only the main findings were highlighted as follows. The Gaussian network was constructed with the normalized scores of each subdimension of the research variables, and the CS-coefficient for the strength of the networks was 0.67, indicating that the centrality stability was excellent.  Figure 2 shows the network of research variables, where the nodes of the same color represent different dimensions of the same variable, solid lines indicate positive correlations among variables, and dashed lines indicate negative correlations among variables. The stronger the correlation among variables, the thicker the line in the graph. The results showed that most variables were correlated. Strong correlations were found between different dimensions of the same variable, except between cynicism and reduced accomplishment. Among different variables, resilience and friend support had the strongest correlation. In addition, resilience, which had the highest bridge centrality strength among all nodes (see Figure 3), suggested that resilience was the bridge node in this network.

## 4. Discussion

The present study indicated that the work experience of male nurses needs to be improved, and further clarified the relative predictive weights of job demands-resources-related variables on work experience. Furthermore, we examined the mutual interactions and structures of PSS, resilience, task load, burnout, and FaW among Chinese male nurses. These findings enriched the research in the occupational health field of male nurses and improved public awareness of the male nurse group.

Compared with previous findings on Chinese nurses [40], the male nurses in this study had slightly lower levels of emotional exhaustion and cynicism and higher levels of low personal accomplishment. It is possible that the current nursing work is not challenging enough for male nurses. Coupled with the influence of traditional concepts, it is not easy for them to be recognized by patients and families, and therefore it is difficult to realize their personal value. The results showed that the FaW score of male nurses was lower than that found in previous studies in the entire nursing population [41]. This indicated that FaW among male nurses still needed to be improved to promote job performance and reduce turnover.

The results of this study suggested that the most important predictor of the occupational burnout of male nurses was PSS. Higher PSS was associated with lower burnout, which was consistent with previous findings [22]. When male nurses receive support from friends, family, and others (e.g., colleagues), their emotional exhaustion reduces and personal accomplishment increases, thereby reducing occupational burnout in clinical practice. In terms of FaW, the dominance analysis showed that PSS and resilience were equally important. Previous research on music teachers and students suggested that work resources such as social support were important antecedents of flow experiences [11]. Csikszentmihalyi's flow theory [42, 43] stated that the fit between the skills and the challenges is a prerequisite for flow to occur. And the study showed that support from colleagues promoted a balance between challenges and skills [11]. Also, the results of a study conducted on athletes showed that brief mindfulness training could significantly increase their flow by improving resilience [26]. This suggests that individuals with higher levels of resilience are more resilient to stress and can easily focus on their work, thus resulting in higher levels of flow. Thus, PSS and resilience, as individual internal and external resources, respectively, might help male nurses recover from frustration more quickly when faced with the constraints of traditional beliefs and work-related pressure, thereby facilitating the generation of flow experiences.

In the present study, the task load had the least effect on both burnout and FaW, which was inconsistent with previous findings [44]. Moreover, the task load negatively correlated with burnout and positively correlated with FaW. According to the job enrichment theory [45, 46], certain job characteristics associated with more complex work and roles, such as increased task diversity and enhanced worker motivation along with positive responses, were expressed as factors associated with increased job satisfaction and reduced burnout. The male nurses in this study might be prone to boredom and burnout due to the high repetition of daily nursing operations and the lack of task richness. Besides, Csikszentmihalyi identified that skill-challenge balance was a prerequisite for flow [47]. In the workplace, employees experienced FaW only when the job requirements and professional skills were balanced and at a high level. This might explain the association of a higher task load with more FaW.

The present study also found resilience important in connecting the final Gaussian network that included all the variables being studied. Both PSS and task load (mainly ME) could affect burnout and FaW through resilience. This suggested a link between nurses' resilience and the ability to maintain healthy psychological characteristics in order to positively cope up with workplace challenges [48]. In addition, higher resilience reduced burnout by promoting FaW (especially work enjoyment). It is possible that male nurses with higher resilience tend to respond more positively when facing various pressures, and hence they were prone to experience FaW. Individuals in the state of flow had clear goals, enjoyed their work, and were unaware of the passage of time [49], which reduced the occurrence of burnout. The results of network analysis also showed the strongest correlation between resilience and friend support among different variables, reflecting that the support from friends was crucial for improving the resilience of male nurses.

This study had some limitations. First, the present findings did not support inferences on the causal relationships among the examined variables or it did not capture the intrapersonal dynamic of the relationships over time because the design of this study was cross-sectional. Second, the study sample included only a part of male nurses from one Chinese province, thus reducing the generalizability of research findings. Furthermore, given the variety of factors that influenced work experience, the findings were likely to be further shaped by certain factors that were not focused on in this survey.

## 5. Conclusions

Male nurses generally have poorer professional recognition and work experience under the influence of many factors such as traditional concepts and stereotypes. Nursing managers should pay attention to the professional development of male nurses and provide them with support at work. At the same time, managers can conduct psychological lectures to improve male nurses' resilience levels. In addition, the results of this study suggest that managers should assign challenging tasks to male nurses based on their individual abilities to leverage their unique strengths.

## 6. Implications for Nursing Management

This study quantitatively compared the relative weights of PSS, resilience, and task load on the burnout and FaW of male nurses using dominance analysis. The findings revealed that PSS was the strongest predictor for occupational burnout among Chinese male nurses, while PSS and resilience were equally important for FaW. The results of the network analysis revealed complex relationships among variables, further suggesting that the interventions aimed at improving the work experience of male nurses could benefit from enhancing resilience. Thus, the interventions might be helpful for nursing managers to take action in order to reduce burnout and to improve FaW among male nurses.

## Figures and Tables

**Table 1:** General information of participants (*n* = 356).

Variables	$\bar{x}$ ± SD (*n*)	Range (%)
Age/years	30.6 ± 5.8	20∼53

*Marital status*		
Married	240	67.4
Other (single or divorced)	116	32.6

*Have child or not*		
Yes	218	61.2
No	138	38.8

*Economic conditions*		
Well	187	52.5
General	92	25.8
Poor	77	21.6

*Educational level*		
Junior college	67	18.8
Undergraduate	260	73.0
Master	29	8.1

*Department*		
Medical ward	45	12.6
Surgical ward	25	7.0
Emergency or intensive care ward	159	44.7
Other	127	35.7

*Professional title*		
Junior	203	57
Intermediate	140	39.3
Senior	13	3.7

*Work position*		
Nurses	308	86.5
Nurse assistant	48	13.5

*Hospital level*		
Tertiary	304	85.4
Secondary and below	52	14.6
Work experience/years	8.5 ± 5.3	1∼32
Monthly night shift/days	7.2 ± 4.5	0∼26

SD, standard deviation.

**Table 2:** Correlation coefficients (rs) among study variables (*n* = 356).

Variables	1	2	3	4	5
(1) Resilience	1				
(2) PSS	0.741^*∗∗*^	1			
(3) Task load	0.259^*∗∗*^	0.266^*∗∗*^	1		
(4) Burnout	−0.364^*∗∗*^	−0.432^*∗∗*^	−0.164^*∗∗*^	1	
(5) Flow at work	0.515^*∗∗*^	0.516^*∗∗*^	0.155^*∗∗*^	−0.438^*∗∗*^	1

*Note.* ^*∗∗*^ *P* means *P* < 0.01. PSS, perceived social support.

**Table 3:** Dominance analysis: average *R*-square across subsets (*n* = 356).

Outcome variable	Number of predictors in the model	PSS	Resilience	Task load
Burnout	0	0.187	0.133	0.027
	1	0.145	0.088	0.012
	2	0.106	0.045	0.002
	General dominance	0.146	0.089	0.014

Flow at work	0	0.266	0.266	0.024
	1	0.177	0.177	0.008
	2	0.101	0.101	∆*R*^2^ < 0.001
	General dominance	0.181	0.181	0.011

PSS, perceived social support.

## Data Availability

The data used to support the findings of this study are available from the corresponding author upon request.
