# Supplementary material for: Work Experience of Chinese Male Nurses Based on the Job Demands-Resources Model: A Cross-Sectional Study
Source: J Nurs Manag. 2023 Feb 21;2023:6486195. doi: 10.1155/2023/6486195 (PMC11918872; doi:10.1155/2023/6486195)
Supplement: Supplementary Materials — The related codes for network analysis are available in the Supplementary File. [file 6486195.f1.docx]

**Supplementary Materials**

**1. Related codes**

*# Load libraries and data*

library(bootnet)

library(qgraph)

library(mgm)

library(psych)

library(haven)

library(networktools)

library(tidyverse)

library(Rcpp)

library(dplyr)

library(corpcor)

data <- read.csv("D:/Desktop/variables.csv", header = TRUE)

attach(data)

*# Rename variables*

short.names <- c("PSS1", "PSS2", "PSS3", "RES", "Load1", "Load2", "Load3", "Load4", "OB1", "OB2","OB3", "FaW1", "FaW2", "FaW3")

long.names <- c("Family support", "Friends support", "Other support", "Resilience", "Mental effort ", "Physical effort", "Time pressure" , "Effort level", "Emotional exhaustion", "Cynicism", "Reduce accomplishment", "Absorption" , "Work enjoyment" , "Intrinsic work motivation" )

groups <- list ("PSS" = c(1,2,3), "Resilience" = c(4), "Task load" = c(5,6,7,8),"OB" = c(9,10,11) ,"FaW" = c(12,13,14))

colour<-list ("PSS" = c(1,2,3), "Resilience" = c(4), "Task load" = c(5,6,7,8),"OB" = c(9,10,11) ,"FaW" = c(12,13,14))

colnames (data) <- short.names

View(data)

*# Estimate network*

ggm <- estimateNetwork (data, default = "EBICglasso", verbose = FALSE)

getWmat(ggm)

adj.ggm <- getWmat(ggm)

round (adj.ggm, 3)

*# plot*

network<- qgraph (adj.ggm, layout = "spring", labels = short.names, nodeNames = long.names,

groups=colour, cut = 0,vsize = 6,border.width = 1.5,theme = "gray", legend.cex = .40)

tiff("Network.tiff", width=6.5, height=4, units="in", res=800, compression="lzw")

qgraph(network, layout = "spring", labels = short.names, nodeNames = long.names, groups = colour, cut = 0,vsize = 6, border.width = 1.5, theme = "gray", negDashed=TRUE, legend.cex = .4)

dev.off()

*# Check stability of the estimates*

boot.edges <- bootnet(ggm, nboots = 2000, nCores = 8)

plot(boot.edges, labels = TRUE, order = "sample")

plot(boot.edges, "edge", plot = "difference", onlyNonZero = TRUE, order = "sample")

tiff("Bootnet - centrality stability.tiff", width=12, height=8, units="in", res=800, compression="lzw")

plot(boot.edges, labels = TRUE, order = "sample")

dev.off()

boot.centrality <- bootnet(ggm, nboots = 3000, nCores = 8, type = "case")

plot(boot.centrality)

tiff("Bootnet - centrality stability.tiff")

plot(boot.centrality)

dev.off()

cs<-corStability(boot.centrality)

write.csv(corStability(boot.centrality), "Stability.csv")

cs

tiff("Bootnet - centrality difference.tiff")

plot(boot.edges, "strength", plot = "difference", order="mean" )

dev.off()

*# Find bridge indicators*

A<-qgraph(ggm$graph, labels=short.names, layout="spring",

vsize=6, cut=0, border.width=12, border.color="black",

groups=groups,

color=c("white", "#E69F00", "#CC79A7", "#009E73", "#56B4E9"),

nodeNames = long.names, legend.cex=.4)

B<-bridge(A, communities = groups, directed=FALSE)

B

tiff("Bridge centrality.tiff", width=4, height=8, units="in", res=800, compression="lzw")

plot(B, include=c("Bridge Strength"), zscore = TRUE)

dev.off()

centrality <- centrality_auto(network, weighted = TRUE, signed = TRUE)

nc <- centrality$node.centrality

SPL <- centrality$ShortestPathLengths

nc

SPL

tiff(file= 'strengh.tiff')

qgraph::centralityPlot(network, include = c("Closeness", "Betweenness", "Strength"), scale = "raw")

dev.off()

tiff("Bridge centrality.tiff", width=4, height=8, units="in", res=800, compression="lzw")

plot(B, include=c("Bridge Strength"), zscore = TRUE)

dev.off()
